# Resource optimization algorithm for RF-aided NOMA VLC network joint blocklength control and power allocation

**DOI:** 10.1371/journal.pone.0353349

**Published:** 2026-07-16

**Authors:** Hongliang Sun, Dejun Xu, Chao Wang, Zhenghua Liao

**Affiliations:** 1 College of Information and Control Engineering, Jilin University of Chemical Technology, Jilin City, Jilin, China; 2 School of Automation Engineering, Northeast Electric Power University, Jilin City, Jilin, China; Federal University of Espirito Santo, BRAZIL

## Abstract

To achieve high-reliability in the Industrial Internet of Things (IIoT) and satisfy the low-latency requirements of industrial equipment, this paper proposes a resource optimization scheme that jointly controls information transmission blocklength and power allocation. Specifically, Short Packet Communication (SPC) and Non-Orthogonal Multiple Access (NOMA) technologies are introduced to construct a Radio Frequency (RF)-aided Visible Light Communication (VLC) network system. The successful transmission probability of multiple User Equipment (UE) is analyzed, and the Service Capacity (SC) of each channel is quantified. Then, an optimization problem is formulated to maximize the SC, subject to constraints on statistical Quality of Service (QoS), Service Reliability (SR), and transmission power. To solve this optimization problem, we design a resource optimization algorithm joint blocklength and power allocation. Simulation results demonstrate that the proposed resource optimization scheme for NOMA VLC/RF networks outperforms NOMA VLC and OMA VLC in ensuring highly reliable data transmission. Furthermore, the proposed algorithm could maximize the SC of the NOMA VLC/RF networks by utilizing shorter blocklength.

## 1. Introduction

The advance of 6G communication would accelerate the comprehensive digital transformation of vertical industries. In the future, the Machine-Type Communication (MTC) within the framework of the Industrial Internet of Things (IIoT) 4.0 would cover different types of equipment in manufacturing, public services, and home management, serving as a key driver of the Fourth Industrial Revolution [1]. IIoT equipment has strict requirements for high-reliability, low-latency, and secure communication [2,3]. Visible Light Communication (VLC), which possesses inherent advantages including abundant spectrum resources, strong immunity to electromagnetic interference, and high security [4–6], could effectively support IIoT equipment connections and improves network throughput.

As a potential technology, Non-Orthogonal Multiple Access (NOMA) could provide the VLC network with higher flexibility and enhance spectrum utilization. The authors of [7] presented a theoretical framework for analyzing the performance of NOMA in a downlink VLC system. Results show that, NOMA can offer a high performance gain in a multi-user VLC system. In addition, the authors of [8] proposed a NOMA-based Light Fidelity (Li-Fi) system, where VLC was used for downlink communication and infrared light was applied to uplink communication to support high-energy efficiency and high-speed IIoT equipment. Furthermore, the authors of [9] considered a downlink multi-user VLC system utilizing NOMA technology, where the NOMA technology was proposed based on individuals and groups, therefore improving user fairness of large-scale equipment accessing the NOMA VLC network. The aforementioned studies demonstrate that applying NOMA technology to the VLC network could improve network performance. However, the VLC network is highly sensitive to blockages, and link interruptions could easily lead to performance degradation. In contrast, Radio Frequency (RF) links have a wider coverage area and stronger diffraction capability, and could serve as an effective supplementary mechanism for the VLC network to improve overall system capacity [10–12].

Applying NOMA technology to VLC/RF hybrid networks could extend the coverage of VLC network while supporting multi-user communication [13,14]. In NOMA VLC network, the authors of [15] employed an RF network as a relay to assist remote users, proposing a weighted energy efficiency indicator to capture the characteristics of the power grid while improving the system’s energy efficiency. Added to that, the authors of [16] constructed a NOMA VLC/RF system based on user mobility. Under incomplete Channel State Information (CSI), the developed system proved that the average energy efficiency of hybrid NOMA VLC/RF systems is better than that of the NOMA VLC system. Furthermore, the authors of [17] exhibited a service mechanism for NOMA VLC/RF networks where strong users could forward messages to weaker ones through VLC/RF links, and designed an adaptive service-oriented user collaboration and link selection strategy to improve system throughput and user reception performance. References [15–17] confirmed that the cooperative mechanism could effectively improve the energy efficiency of hybrid NOMA VLC/RF networks. However, when supporting Ultra-Reliable Low-Latency Communications (URLLC) equipment requirements, traditional long packet transmission technology increases end-to-end latency due to the large data processing and encapsulation overhead. In contrast, Short Packet Communication (SPC) technology is a promising URLLC technology that could enhance system reliability by transmitting smaller data units.

To more efficiently support URLLC requirements, SPC has been proposed for applications within wireless communication network [18]. For instance, the authors of [19] studied the SPC of the multiple-input multiple-output-NOMA two-hop relay systems and derived a closed form expression for the asymptotic block error rate. Simulation results showed that the systems met the requirements of URLLC. The authors of [20] used the Full-Duplex (FD)-cooperative NOMA SPC systems to forward information toward remote users. Compared to long-packet communication, FD C-NOMA-SPC yielded a narrower power allocation range and could achieve highly reliable transmission. Furthermore, the authors of [21] designed a NOMA system with SPC enhancement by allocating different powers to User Equipment (UE), where all users could get reliable communication at the same time. References [18–21] demonstrated that SPC technology could effectively support URLLC services, providing theoretical support for its application in the constructed RF-aided NOMA-VLC network. In wireless communication networks, the achievable instantaneous rate varies randomly. Therefore, it is challenging to ensure delay performance for delay-sensitive equipment while enhancing Service Reliability (SR).

Motivated by the above challenges, we propose a new resource optimization scheme that jointly controls blocklength and power allocation for the NOMA VLC/RF scenario. The proposed solution could efficiently guarantee the differentiated Quality of Service (QoS) requirements of multi-UE while enhancing the Service Capacity (SC) and SR. The main contributions of this work are as follows:

- An RF-aided NOMA VLC transmission model is established. Utilizing the SPC technology, the successful transmission probability of multi-UE is calculated, and we obtain an instantaneous service expression. Furthermore, the SC is quantified based on the effective capacity theory.
- Under the constraints of statistical delay QoS, SR, and transmission power, the optimization problem of maximizing SC is devised. To solve this problem efficiently, we propose a resource optimization algorithm combining blocklength control and power allocation.

## 2. NOMA VLC/RF networks model

**Fig 1:**
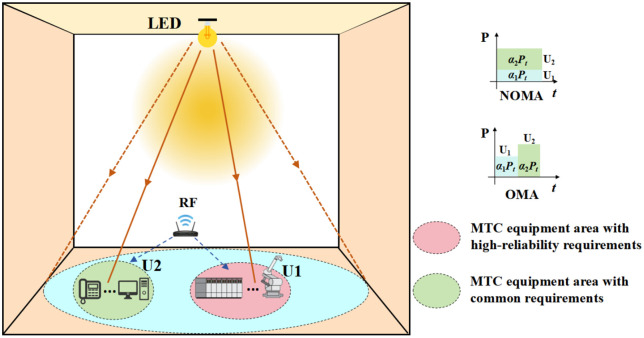
NOMA VLC/RF networks model.

In this paper, we analyze the NOMA VLC/RF downlink networks containing *K* MTC equipment. The specific scenario is illustrated in Fig 1. The VLC network enables data transmission through intensity modulation of Light Emitting Diode (LED) light sources. According to the different QoS requirements of UE, the UE in VLC/RF networks is classified into two categories: the number of MTC equipment with high-reliability and common demands is *X* and *Y*, respectively. There is an RF Access Point (AP) at the same height as the UE, and the VLC AP is placed in the center of the roof. The UE equipped with Photo Detectors (PD) communicates with the VLC network preferentially. If and only if the UE fails to decode the VLC signal, the RF network could continue to communicate with the UE as a compensation scheme. RF could serve multi-UE simultaneously. It is assumed that the channel is static or quasi-static. Therefore, the UE does not change the state of the channel during each transmission, and the CSI is completely known. As the diffusion non-line-of-sight link component is basically ignored, only the line-of-sight link component is considered in this work [22]. To achieve low-latency communication, each UE employs SPC technology to transmit the signal.

Each UE in the VLC/RF networks has differentiated QoS requirements. The QoS level of each category considers the highest QoS requirements of the UE within the group. From a statistical perspective, the *i*-th MTC equipment must satisfy the probability $Pr\{d_{i}>D_{i}^{max}\}\approx e^{-\theta_{i}\mu_{i}D_{i}^{max}}$, where *d*_*i*_ and $D_{i}^{max}$ denote the steady-state and maximum tolerable delay, respectively. Moreover, *θ*_*i*_ is the QoS index, and *μ*_*i*_ represents the average arrival rate [23]. For a particular $D_{i}^{max}$, a larger QoS index *θ*_*i*_ corresponds to a stricter delay QoS constraint. To more clearly explain the signal transmission process of the NOMA VLC/RF networks, the following part selects *X* = 1 and *Y* = 1 as an example for research without loss of generality. Consequently, the equipment with high-reliability requirements and the one with common requirements are represented by UE_1_ and UE_2_, respectively.

### 2.1. Signal transmission process with NOMA VLC/RF networks

In the NOMA VLC/RF networks model constructed in this paper, UE_*i*_ (*i*∈{1, 2}) will send signal to both the VLC AP and RF AP at the same time. It is worth noting that UE_*i*_ can only receive the VLC or RF signal, but not both signals simultaneously. When the VLC signal is successfully decoded, the VLC, as the primary communication network, provides service to UE_i_ through NOMA technology. Conversely, if the VLC signal is incorrectly decoded, the RF network could be regarded as a compensation solution.

#### 2.1.1. VLC signal transmission process based on NOMA technology.

In the adopted intensity modulation and direct detection scheme, the VLC network requires that the transmitted signal is real and non-negative. To generate real-valued signal, Hermitian symmetry is typically applied. Additionally, a DC bias is introduced at the LED to ensure the non-negativity of the transmitted signal. The superimposed signal received at the VLC AP is expressed as follows:


$$\begin{array}{c} x_{\text{VLC}}=\sqrt{\alpha_{\text{1}}P_{t}}S_{\text{1}}+\sqrt{\alpha_{\text{2}}P_{t}}S_{\text{2}}+I_{\text{DC}}, \end{array}$$ (1)

where *P*_*t*_ represents the VLC transmission power, *I*_DC_ denotes the DC bias of the signal, *S*_*j*_ (*j*∈{1,2}) is the information signal sent by VLC to UE_*i*_ (*i.e., S*_1_ → UE_1_, *S*_2_ → UE_2_), and *α*_1_ and *α*_2_ are the power coefficients of the signal assigned to UE_*i*_.

After the optical-to-electrical power conversion and the elimination of the DC bias at the receiver, the received signal at the UE_*i*_ is expressed as follows:


$$\begin{array}{c} y_{i}=h_{i}^{VLC}\sum {\mathrm{\backslash nolimits}}_{i=1}^{K}\sqrt{\alpha_{i}P_{t}}S_{j}+n_{i}, \end{array}$$ (2)

where $n_{i}\;\sim CN\left(0,\sigma_{VLC}^{\text{2}}\right)$ represents the VLC network noise, which is considered to be Additive White Gaussian Noise (AWGN) with zero values for both mean and variance. It is calculated using $\sigma_{VLC}^{\text{2}}=N_{V0}⬝B_{V}$ [24]. Moreover, *B*_*V*_ denotes the VLC network bandwidth, and *N*_*V*0_ is the noise power spectral density of the VLC network. Finally, $h_{i}^{VLC}$ is the channel gain between VLC AP and UE_*i*_, formulated as follows:


$$\begin{array}{c} h_{i}^{VLC}=\left\{ \begin{array}{c} @l@\frac{(\delta +1)A_{\text{c}}R_{\text{0}}}{2\pi \lambda_{i}^{\text{2}}}{cos}^{m}\left(\phi_{i}\right)T\left(\psi_{i}\right)g\left(\psi_{i}\right)cos\left(\psi_{i}\right),\;\text{0}\leq \psi_{i}\leq \Psi_{\text{FOV}}\text{,}\\ 0,\;\psi_{i}>\Psi_{\text{FOV}}\text{\textbackslash{}hspace\{0.5em}\}, \end{array}\right.\text{\textbackslash{}hspace\{0.5em}\;\} \end{array}$$ (3)

where $\delta ≜-ln2/ln\left(cos\left(\Phi_{1/2}\right)\right)$ is the Lambert coefficient, and $\Phi_{1/2}$ represents the half-power angle of the LED. Moreover, *A*_c_ and *R*_0_ are the receiving area and optical-to-electrical power conversion coefficient of PD, respectively. In addition, $\phi_{i}$ and $\psi_{i}$ represent the emission angle of the LED and the incident angle of the optical detector PD at the receiving end, respectively, whereas $\Psi_{\text{FOV}}$ expresses the field of view of PD. In addition, λ_*i*_ is the straight-line distance between VLC AP and PD. Finally, $T\left(\psi_{i}\right)$ and $g\left(\psi_{i}\right)$ represent the gain of the optical filter and optical concentrator at the receiver, respectively.

At UE_2_, *S*_1_ is considered as interference, and *S*_2_ is decoded directly. The Signal to Interference Noise Ratio (SINR) corresponding to the UE_2_ decoded signal *S*_2_ is expressed as follows:


$$\begin{array}{c} r_{22}=\frac{\alpha_{\text{2}}P_{t}H_{\text{2}}}{\alpha_{\text{1}}P_{t}H_{\text{2}}+1}, \end{array}$$ (4)

where $H_{i}≜\frac{(R_{\text{0}}h_{i}^{VLC}{)}^{\text{2}}}{\sigma_{VLC}^{\text{2}}}$ represents the normalized channel gain of UE_*i*_. At UE_1_, the receiver first performs the Successive Interference Cancellation (SIC) and decodes *S*_2_. After successful decoding, *S*_2_ is subtracted from the total signal, then *S*_1_ is detected and decoded. Thus, the SINR expressions corresponding to the UE_1_ decoded signals *S*_2_ and *S*_1_ are defined as follows:


$$\begin{array}{c} r_{12}=\frac{\alpha_{\text{2}}P_{t}H_{\text{1}}}{\alpha_{\text{1}}P_{t}H_{\text{1}}+1}, \end{array}$$ (5)

$$\begin{array}{c} r_{11}=\alpha_{\text{1}}P_{t}H_{\text{1}}. \end{array}$$ (6)

#### 2.1.2. RF signal transmission process.

The transmitted signal *x*_*Ri*_ of UE_*i*_ at the RF AP can be written as follows:


$$\begin{array}{c} x_{Ri}=\sqrt{P_{R}}S_{j}^{RF}, \end{array}$$ (7)

$S_{j}^{RF}$ is the information signal sent by RF to UE_*i*_. Moreover, *P*_*R*_ represents the RF transmission power.

The signal *y*_*Ri*_ received by UE_*i*_ satisfies the following relation:


$$\begin{array}{c} y_{Ri}=h_{i}^{RF}x_{R}+n_{R}, \end{array}$$ (8)

$n_{R}$ represents AWGN where its mean and variance are set to zero and $\sigma_{RF}^{\text{2}}$, respectively. Moreover, $h_{i}^{RF}$ is the channel gain between RF and UE_*i*_, and it is expressed as follows:


$$\begin{array}{c} h_{i}^{RF}=10^{\frac{-L\left(\lambda_{Ri}\right)}{10}}{\left|h_{r}\right|}^{\text{2}}, \end{array}$$ (9)

where *λ*_*Ri*_ represents the straight-line distance from RF to UE_*i*_, *h*_*r*_ expresses the fading channel, being an exponential random variable with a mean of 2.46 dB, and *L*(*λ*_*Ri*_) is the path propagation loss expressed as follows:


$$\begin{array}{c} L\left(\lambda_{Ri}\right)=47.9+10\times v\times lg(\lambda_{Ri}/\lambda_{R0})+𝕏, \end{array}$$ (10)

where *v* is the path loss factor. In addition, *λ*_*R*0_ = 1/m is the reference distance. The shadow component is denoted by 𝕏, which is a Gaussian random variable with a zero mean and variance equal to 1.8 dB.

The SINR expression corresponding to the UE decode RF signal is expressed as follows:


$$\begin{array}{c} r_{Ri}=\frac{P_{R}(h_{i}^{RF}{)}^{\text{2}}}{N_{R0}B_{R}}, \end{array}$$ (11)

where *N*_*R*0_ expresses the noise power spectral density of the RF [17]. *B*_*R*_ denotes the RF channel bandwidth.

### 2.2. VLC signal transmission process based on OMA technology

To demonstrate the advantages of NOMA technology, this paper selects OMA as the comparison scheme of NOMA to implement the solution proposed. Through OMA, it is assumed that both UEs are distinguished by the time division multiple access technique to prevent interference between UEs. At UE_*i*_, the transmitted signal can be written as follows:


$$\begin{array}{c} x_{i}^{o}=\sqrt{\alpha_{i}P_{t}}S_{j}+I_{DC}. \end{array}$$ (12)

The received signal of UE_*i*_ in the OMA solution is expressed as follows:


$$\begin{array}{c} y_{i}^{o}=h_{i}^{o}\sqrt{\alpha_{i}P_{t}}S_{j}+n_{i}. \end{array}$$ (13)

Finally, the SINR expression of the UE_*i*_ decoded signal is denoted as follows:


$$\begin{array}{c} r_{i}^{o}=\alpha_{i}P_{t}H_{i}. \end{array}$$ (14)

## 3. Design of resource optimization algorithm

### 3.1. Service Capability evaluation of NOMA VLC/RF networks

According to reference [25], short packet transmission technology is used in NOMA VLC/RF networks. Thus, the instantaneous bit per channel use (BPCU) $R_{i}^{\mathbb{N}}\text{(}\mathbb{N}\in \left\{\text{VLC,\textasciitilde{}RF}\right\})$ of UE_*i*_ can be written as follows:


$$\begin{array}{c} R_{i}^{\mathbb{N}}={𝕎}^{VLC}\frac{k}{L_{i}^{VLC}}+{𝕎}^{RF}\frac{k}{L_{i}^{RF}}, \end{array}$$ (15)

where ${𝕎}^{VLC}\in \left\{0,1\right\}$ and ${𝕎}^{RF}\in \left\{0,1\right\}$ are the selection variables of VLC network and RF network, respectively. Moreover, $L_{i}^{VLC}$ and $L_{i}^{RF}$ denote the blocklength of VLC network and RF network, respectively, and *k* represents the number of transmitted bits of UE_*i*_.

For VLC/RF networks, at a given instantaneous transmission rate $R_{i}^{\mathbb{N}}$ and blocklength $L_{i}^{\mathbb{N}}$, the decoding error probability of signal *S*_*j*_ at UE_*i*_ can be approximated as follows:


$$\begin{array}{c} \varepsilon_{ij}^{\mathbb{N}}\approx Q\left(f_{\mathbb{N}}\left(r_{ij}^{\mathbb{N}},L_{i}^{\mathbb{N}},R_{i}^{\mathbb{N}}\right)\right), \end{array}$$ (16)

where the Gaussian function *Q* is $Q\left(x\right)=\int_{x}^{\infty }\sqrt{1/(2\pi )}e^{-t^{\text{2}}/2}dt$. $r_{ij}^{\mathbb{N}}={𝕎}^{VLC}r_{ij}\text{+}{𝕎}^{RF}r_{Ri}$, *i*, *j*∈{1,2}.

The function $f_{\mathbb{N}}(\cdot )$ can be obtained using the following:


$$\begin{array}{c} f_{\mathbb{N}}\left(r_{ij}^{\mathbb{N}},L_{i}^{\mathbb{N}},R_{i}^{\mathbb{N}}\right)≜{𝕎}^{VLC}\left(\frac{ln2\sqrt{L_{i}^{VLC}}(0.5{log}_{\text{2}}(1+r_{ij})-R_{i}^{VLC})}{\sqrt{0.5(1-(1+r_{ij}{)}^{-2})}}\right)+{𝕎}^{RF}\left(\frac{ln2\sqrt{L_{i}^{RF}}\left({log}_{\text{2}}\left(1+r_{Ri}\right)-R_{i}^{RF}\right)}{\sqrt{\left(1-(1+r_{Ri}{)}^{-2}\right)}}\right). \end{array}$$ (17)

The NOMA technology transmits signal to two UE simultaneously with different power allocations, so NOMA can serve UE_1_ and UE_2_ (i.e., $L_{\text{1}}^{VLC}=L_{\text{2}}^{VLC}=L^{VLC}$) at the same time. In the NOMA VLC network, according to Eq. (16), the decoding error probability of *S*_2_ at UE_2_ is approximately:


$$\begin{array}{c} \varepsilon_{22}^{VLC}\approx Q\left(f_{VLC}\left(r_{22},L_{\text{2}}^{VLC},R_{\text{2}}^{VLC}\right)\right). \end{array}$$ (18)

For UE_2_, only *S*_2_ should be decoded. There is only one decoding situation at UE_2_. As a result, the probability of successful access of UE_2_ is expressed as follows:


$$\begin{array}{c} p_{s,2}^{VLC}=1-\varepsilon_{22}^{VLC}. \end{array}$$ (19)

For UE_1_, it is necessary to decode *S*_2_ first and then decode *S*_1_. The decoding error probability of *S*_2_ and *S*_1_ at UE_1_ is defined as follows:


$$\begin{array}{c} \varepsilon_{12}^{VLC}\approx Q\left(f_{VLC}\left(r_{12},L_{\text{1}}^{VLC},R_{\text{1}}^{VLC}\right)\right),\;\varepsilon_{11}^{VLC}\approx Q\left(f_{VLC}\left(r_{11},L_{\text{1}}^{VLC},R_{\text{1}}^{VLC}\right)\right). \end{array}$$ (20)

UE_1_ is successfully accessed if and only if both signals *S*_1_ and *S*_2_ are decoded successfully. Then, the probability of successful access of UE_1_ is expressed as follows:


$$\begin{array}{c} p_{s,1}^{VLC}=1-\left[\varepsilon_{12}^{VLC}+\left(1-\varepsilon_{12}^{VLC}\right)\varepsilon_{11}^{VLC}\right]. \end{array}$$ (21)

When the UE fails to decode the VLC signal, the RF network provides service to the UE. Currently, the decoding error probability of $\varepsilon_{i}^{RF}$ (*i*∈{1,2}) at UE_*i*_ is defined as follows:


$$\begin{array}{c} \varepsilon_{i}^{RF}\approx Q\left(f_{RF}\left(r_{Ri},L_{i}^{RF},R_{i}^{RF}\right)\right). \end{array}$$ (22)

Moreover, the probability of successful access of UE_*i*_ is expressed as follows:


$$\begin{array}{c} p_{s,i}^{RF}=1-\varepsilon_{i}^{RF}. \end{array}$$ (23)

In the OMA VLC network, according to Eq. (16), since both UEs are assigned to different orthogonal resources, the probability of successfully access UE_*i*_ is defined as follows:


$$\begin{array}{c} p_{s,i}^{o}=1-\varepsilon_{i}^{VLC}. \end{array}$$ (24)

Due to the decoding error probability, the rate matched by the network for the UE is different. The VLC signal is decoded successfully, $\begin{array}{cc} {𝕎}^{VLC}=1, & {𝕎}^{RF}=0 \end{array}$, the UE_*i*_ will get the service rate $R_{i}^{VLC}$ provided by the VLC network. However, the VLC signal decoding error occurs and the RF signal decoding is successful, $\begin{array}{cc} {𝕎}^{VLC}=0, & {𝕎}^{RF}=1 \end{array}$, the RF network allocates the service with a rate of $R_{i}^{RF}$ to the UE. Finally, when both the VLC signal and the RF signal are decoded incorrectly, $\begin{array}{cc} {𝕎}^{VLC}=0, & {𝕎}^{RF}=0 \end{array}$, the actual data rate transmitted by the network is set to zero. Considering the relationship between network rate and the probability of successfully access, the instantaneous service model in bits per $L_{i}^{\mathbb{N}}$ channel use can be described as follows:


$$\begin{array}{c} s_{i}\left(n\right)=\left\{ \begin{array}{c} @l@\begin{array}{cc} @llL_{i}^{VLC}R_{i}^{VLC}, & \;p_{s,i}^{VLC},\mathrm{\backslash vspace}1mm\\ L_{i}^{RF}R_{i}^{RF}, & (1-p_{s,i}^{VLC})p_{s,i}^{RF}, \end{array}\mathrm{\backslash vspace}1mm\\ 0,\;(1-p_{s,i}^{VLC})(1-p_{s,i}^{RF}).\text{\textbackslash{}hspace\{0.5em}\} \end{array}\right. \end{array}$$ (25)

Based on reference [23], this paper quantifies the per channel SC provided by the NOMA-VLC/RF networks to UE_*i*_ (*i*∈{1,2}) as follows:


$$\begin{array}{c} SC_{i}\left(\theta_{i}\right)=-\frac{ln\left[p_{s,i}^{VLC}e^{-\theta_{i}L_{i}^{VLC}R_{i}^{VLC}}+(1-p_{s,i}^{VLC})p_{s,i}^{RF}e^{-\theta_{i}L_{i}^{RF}R_{i}^{RF}}+\left(1-p_{s,i}^{VLC}\right)\left(1-p_{s,i}^{RF}\right)\right]}{\theta_{i}\left(p_{s,i}^{VLC}L_{i}^{VLC}+\left(1-p_{s,i}^{VLC}\right)L_{i}^{RF}\right)}. \end{array}$$ (26)

### 3.2. Construction and solution of the SC maximization problem

#### 3.2.1. Optimization problem construction.

Reliability is an important indicator to measure the performance of the communication network. This paper defines SR at UE_*i*_ (*i*∈{1,2}) the probability that a data packet is successfully decoded. The SR at UE_*i*_ can be described as follows:


$$\begin{array}{c} \left\{ \begin{array}{c} @l@\chi_{i}^{VLC/RF}=p_{s,i}^{VLC}\cdot 100(\%)+(1-p_{s,i}^{VLC})p_{s,i}^{RF}\cdot 100(\%),\\ \chi_{i}^{VLC}=p_{s,i}^{VLC}\cdot 100(\%). \end{array}\right. \end{array}$$ (27)

Since VLC network offers higher data rates than RF network over short distances, it present broad application prospects in wireless communication scenarios [5]. Based on this, we focus on the joint optimization problem of blocklength control and power allocation, under the premise of fixed RF network parameters. The objective is to maximize the SC of NOMA VLC/RF networks, while ensuring statistical delay QoS requirements and SR in multi-UE network. The optimization problem can be expressed as follows:


$$\begin{array}{c} \begin{array}{c} \;\underset{\left\{\alpha_{\text{1}},\alpha_{\text{2}},L_{i}^{VLC}\right\}}{max}\sum_{i=1}^{\text{2}}SC_{i}\left(\theta_{i}\right)\mathrm{\backslash vspace}1mm\\ s.t.\text{ C1: }\alpha_{\text{1}}+\alpha_{\text{2}}\leq 1,\mathrm{\backslash vspace}1mm\\ \text{ C2}:0<\alpha_{\text{1}}<\alpha_{\text{2}},\mathrm{\backslash vspace}1mm\\ \text{ C3}:L_{min}^{VLC}\leq L_{i}^{VLC}\leq L_{max}^{VLC}\text{,}L_{i}^{VLC}\in {\mathbb{Z}}^{+},\mathrm{\backslash vspace}1mm\\ \text{ C4: }EB_{i}\left(\theta_{i}\right)\leq SC_{i}\left(\theta_{i}\right),i\in \left\{1,2\right\},\mathrm{\backslash vspace}1mm\\ \text{ C5: }min\left(\chi_{\text{1}}^{VLC/RF},\chi_{\text{2}}^{VLC/RF}\right)\geq \chi_{th}^{VLC/RF}, \end{array} \end{array}$$ (28)

where effective bandwidth, denoted as $EB_{i}\left(\theta_{i}\right)$, is defined as the minimum bandwidth resource required to support arrival services while satisfying the fixed QoS requirements. Moreover, $\chi_{th}^{VLC/RF}$ represents the target SR in VLC/RF network, ${\mathbb{Z}}^{+}$ denotes a set of positive integers, $L_{min}^{\mathbb{N}}$ and $L_{max}^{\mathbb{N}}$ highlight the minimum and maximum blocklength of the VLC network, respectively. Moreover, C1 and C2 represent power constraints, C3 expresses the VLC blocklength constraint, C4 denotes the network statistical delay QoS constraint, and C5 is the SR constraint in VLC/RF networks.

#### 3.2.2. Motivation for designing the solution.

The convex optimization is a typical approach to settle the optimization problem. The objective function constructed in this paper incorporates multiple nonlinear components, such as the Q-function, logarithmic function, and exponential function. As a result, obtaining its derivative is not easy, which would increase the difficulty of analyzing its concavity or convexity.

The heuristic-based approaches offer an alternative for settling optimization problems. The solution process relies on extensive stochastic search and parameter adjustment, resulting in relatively high execution cost. Consequently, we propose a resource optimization algorithm for joint blocklength control and power allocation based on grid search, thereby solving this optimization problem while reducing the complexity of model analysis.

#### 3.2.3. Analyzing boundaries of VLC power coefficients and blocklength allocation.

In the process for settling optimization problems utilizing search mechanisms, the undefined search boundaries often lead to a large amount of computation. Therefore, we need to calculate the bounds of the power coefficients and the VLC blocklength to compress the search space, thereby improving the computational efficiency.

Due to the SIC technology characteristics, as long as the SR of UE_1_ can be guaranteed, the SR of UE_2_ in VLC network can also have the same guarantee. Therefore, the SR in the VLC network should be met $\chi_{\text{1}}^{VLC}\geq \chi_{th}^{VLC}$, where $\chi_{\text{1}}^{VLC}$ and $\chi_{th}^{VLC}$ denote the SR and the target SR in the VLC network, respectively. Since the decoding error probability of VLC signal is a monotonically decreasing function of SINR [26], when defining the blocklength, the SR at UE_1_ is met with the lowest standard. That is, when $\chi_{\text{1}}^{VLC}=\chi_{th}^{VLC}$, the minimum resource configuration can be generated. According to Eq. (21), using the appropriate scaling, we can obtain:


$$\begin{array}{c} min\left(p_{s,1}^{VLC}\right)=1-\varepsilon_{11}^{VLC}>1-\left[\varepsilon_{12}^{VLC}+\left(1-\varepsilon_{12}^{VLC}\right)\varepsilon_{11}^{VLC}\right]=\frac{\chi_{th}^{VLC}}{100}. \end{array}$$ (29)

Thus, the lower bound of the SINR of *S*_1_ decoded at UE_1_ can be obtained as follows:


$$\begin{array}{c} r_{11}^{min}=f_{VLC}^{\;-1}(Q^{-1}(1-min\left(p_{s,1}^{VLC}\right)),L_{\text{1}}^{{VLC}^{*}},R_{\text{1}}^{{VLC}^{*}}). \end{array}$$ (30)

According to Eq. (6), we can further obtain:


$$\begin{array}{c} \alpha_{\text{1}}^{LB}=\frac{r_{11}^{min}}{P_{t}H_{\text{1}}}. \end{array}$$ (31)

The rate of short packet transmissions is smaller than the Shannon rate; thus, the SINR of UE_*i*_ needs to satisfy $r_{i}^{min}>{\text{2}}^{R_{VLC,i}^{min}}-1$.

According to the theory of effective bandwidth and effective capacity, the statistical delay QoS of UE_*i*_ is guaranteed if and only if the effective capacity is greater or equal to the effective bandwidth [27]. Changing C3 to $SC_{i}\left(\theta_{i}\right)=EB_{i}\left(\theta_{i}\right)$ can yield that when the UE is in a VLC network, the minimum rate requirements that can guarantee the statistical delay QoS is denoted as follows:


$$\begin{array}{c} R_{VLC,i}^{min}=-\frac{\text{1}}{\theta_{i}L_{i}^{VLC}}ln\left(\frac{e^{-\theta_{i}EB_{i}\left(\theta_{i}\right)\left(p_{s,i}^{VLC}L_{i}^{VLC}+\left(1-p_{s,i}^{VLC}\right)L_{i}^{RF}\right)}-(1-p_{s,i}^{VLC})p_{s,i}^{RF}e^{-\theta_{i}L_{i}^{RF}R_{i}^{RF}}-(1-p_{s,i}^{VLC})(1-p_{s,i}^{RF}))}{p_{s,i}^{VLC}}\right). \end{array}$$ (32)

Thus, the power coefficient by the VLC network to UE_1_ is transformed into:


$$\begin{array}{c} \alpha_{\text{1}}\text{'}=\frac{P_{t}H_{\text{2}}-({\text{2}}^{R_{VLC,2}^{min}}-1)}{{\text{2}}^{R_{VLC,2}^{min}}P_{t}H_{\text{2}}}. \end{array}$$ (33)

According to Eq. (33) and constraint C2, the upper bound of the power coefficient of the VLC network to UE_1_ is expressed as follows:


$$\begin{array}{c} \alpha_{\text{1}}^{UB}=min[\frac{P_{t}H_{\text{2}}-({\text{2}}^{R_{VLC,2}^{min}}-1)}{{\text{2}}^{R_{VLC,2}^{min}}P_{t}H_{\text{2}}},0\mathrm{.5}]. \end{array}$$ (34)

Similarly, when defining the power allocation scheme, the lower bound on the blocklength for VLC network can be derived by meeting the SR requirements at UE with the minimum permissible standard, expressed as follows:


$$\begin{array}{c} L_{\min}^{VLC}=f_{VLC}^{\;-1}(Q^{-1}(1-\min\left(p_{s,1}^{VLC}\right)),r_{11}^{*},R_{\text{1}}^{{VLC}^{*}}). \end{array}$$ (35)

#### 3.2.4. Solving optimization problem.

Within the derived bounds of blocklength and power coefficient, a two-layer one-dimensional linear search is employed to determine the optimal set of blocklengths $L^{VLC*}$ and power coefficients *α*_1_* and *α*_2_* that maximize the SC. The specific algorithm process is as follows:

**Algorithm 1 Resource optimization algorithm for joint blocklength control and power allocation**

**Input: *θ***_*i*_, *EB*_*i*_ (***θ***_*i*_), $\chi_{th}^{VLC/RF}$, *P*_*R*_, *P*_*t*_, $L_{i}^{RF}$, *k* = 0.

**Output:** VLC network optimal blocklength $L^{VLC*}$, optimal power allocation coefficients *α*_1_*, *α*_2_* for VLC network.

1: Calculate the SINR *r*_R1_, *r*_R2_ of UE_1_ and UE_2_ under the RF network

2: Calculate the corresponding rate $R_{i}^{RF}$, and the probability of successful transmission of the RF signal $p_{s,i}^{RF}$

3: **For** *n* ← 1 *to* length(*α*_1_) **do**// *α*_1_=$\alpha_{\text{1}}^{LB}:\kappa :\alpha_{\text{1}}^{UB}$, from (34) and (31), get the upper and lower bounds of *α*_1_

4:   According to *α*_1_(*n*), the SINR *r*_11_(*n*), *r*_12_(*n*), *r*_22_(*n*) of UE_1_ and UE_2_ in the VLC network are calculated

5:   **For** *m* ← 1 *to* length($L^{VLC}$) **do**// $L^{VLC}$ =$L_{min}^{VLC}:\vartheta :M\cdot L_{min}^{VLC}$, from (35), Get $L^{VLC}$ lower bound

6:     Calculate the rate $R_{i}^{VLC}$ (*n*,*m*) and signal $p_{s,i}^{VLC}$ (*n*,*m*) in the VLC network based on $L^{VLC}$ (*m*)

7:     Calculate the *SC*_*i*_(*n*,*m*) of each UE according to the ***θ***_*i*_, and calculate the *SC*_*sum*_(*n*,*m*)

8:     **If** (SC_*i*_(*n*,*m*) ≥ *EB*_*i*_(***θ***_*i*_)) && $\left(\chi_{\text{1}}^{VLC/RF}\geq \chi_{th}^{VLC/RF}\right)$ **then**

9:      **O**(*k*,:) = [*SC*_*sum*_(*n,m*), *α*_1_(*n*), $L^{VLC}$ (*m*)]; *k* = k + 1;

10:     **End If**

11:   **End For**

12: **End For**

13: OP* = *arg max*{**O**(:, 1)}

14: **Return** OP* = [*SC*_sum_*, *α*_1_*, $L^{VLC*}$, *α*_2_*=1-*α*_1_*]

The search interval covers the entire feasible solution space, and all feasible solutions lie within this interval. For each pair (*α*_1_, $L^{VLC}$), the corresponding objective function value is determined, and the constraint satisfaction is verified. The proposed algorithm could maximize the sum SC while ensuring the requirements of statistical QoS. It merely requires a sequential traversal of all discrete values to obtain the optimal solution without requiring real-time response. Compared with heuristic-based approaches, the algorithm avoids the potential issue of getting trapped in local optima, thereby ensuring global optimality.

Next, we analyze the complexity of the proposed resource optimization algorithm. The complexity mainly stems from the maximum number of points searched in two one-dimensional search layers, which is determined by the search step sizes κ (for the power coefficient) and ϑ (for the blocklength). Let $\xi_{\text{1}}=length(\alpha_{\text{1}}^{LB}:\kappa :\alpha_{\text{1}}^{UB})$ denote the number of search points for the power coefficient, and $\xi_{\text{2}}=length\left(L_{min}:\vartheta :\left(M\cdot L_{min}\right)\right)$ is the number of search points for the blocklength. Consequently, the overall algorithmic complexity is O($\xi_{\text{1}}$ ×$\xi_{\text{2}}$). The value of the step sizes κ and ϑ could affect the accuracy and computational cost of the proposed algorithm. The smaller step sizes yield a denser grid and thus more accurate solutions near the optimum. This comes at the cost of higher computational load. The parameter *M* defines the search range for the blocklength, and its value could be determined based on system constraints to ensure that the optimal solution lies within the interval, and a larger *M* increases the computational burden.

### 3.3. Generalization to Multi-UE Scenario

For the SPC-NOMA-VLC/RF model containing *K* users (*K* = *X* + *Y*), it is necessary to reanalyze the signal decoding error probability and reassess the SC of VLC/RF networks. The specific steps are as follows:

(1) **QoS grouping**. UE should be classified according to QoS requirements to determine the value of the QoS index within each group.
(2) **Signal Decoding Process in the VLC/RF systems.** The *k*-th user (UE_*k*_) utilizes SIC to recover its own signal *S*_*k*_. This process requires UE_*k*_ to sequentially decode stronger signals until all signals are decoded or the remaining signal is too weak to decode. Therefore, the condition for successfully decoding the *j*-th user’s message (*j* ≤ *k*) is that the corresponding SINR satisfies:

$$\begin{array}{c} r_{k\text{\textbackslash{}hspace\{0.5em}}j\}=\frac{\alpha_{j}P_{t}H_{k}}{\sum_{i=j+1}^{K}\alpha_{i}P_{t}H_{k}+1} \end{array}$$ (36)

where the $\sum_{i=j+1}^{K}\alpha_{i}P_{t}H_{k}$ in the denominator represents the residual inter-user interference.

If the VLC transmission for UE_*k*_ fails, the system switches to the RF link. A notable point is that the presence of multi-UE does not affect the decoding process in the RF link.

(3) **Calculate the probability of successfully access the VLC channel**. For the *k*-th user, the condition for successful communication is that each of the signals *S*_1_, *S*_2_,..., *S*_*k*_ is entirely decoded. Therefore, the probability of successfully access for VLC is the product of the success probabilities of all steps.
(4) **SC Evaluation for the Multi-UE System**. The SC of the multi-UE VLC/RF networks could be evaluated directly using the framework established in Eq. (26), on the premise of reconstruction the instantaneous service rate model for multi-UE based on Eq. (25).

## 4. Simulation results and analysis

The performance of the proposed resource optimization algorithm is assessed through MATLAB platform, and the computer is equipped with AMD Ryzen 7 8845H processor, 32 GB RAM, and NVIDIA GeForce RTX 4060 GPU. The simulation parameters are shown in Table 1, where the specific values are set referring to [14,28–30].

**Table 1:** Simulation parameter table in VLC/RF networks.

Parameters	Values
LED half power angle $\Phi_{1/2}$	60°
Coverage of LED *r*_*c*_	3 m
PD field of view $\Psi_{\text{FOV}}$	60°
Receiving area of PD *A*_*c*_	1 cm^2^
optical-to-electrical conversion coefficient of PD *R*_0_	0.53 A/W
VLC network noise *N*_*V*0_	10^-13^ A^2^/Hz
Number of bits of information transmitted *k*	50 bit
Path loss factor *v*	0.2
RF network noise *N*_*R*0_	-86dBm/Hz
RF transmission power *P*_*R*_	5 dBm

**Fig 2:**
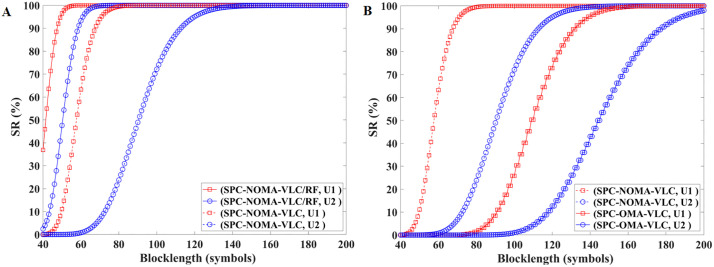
The impact of blocklength on the SR under different solutions. (A) Different scenarios. (B) Different technologies.

First, to prove the superiority of the proposed scheme, SPC is applied in the NOMA VLC/RF, NOMA VLC, and OMA VLC networks, and the resource optimization schemes are designed. Fig 2 illustrates the impact of the use of various schemes on SR. The VLC transmission power *P*_*t*_ is set to 15 dBm, and the blocklength ranges between 40 and 200 symbols. As illustrated in Fig 2, the SR increases with the blocklength. This is because when the blocklength increases, the decoding error probability becomes smaller, thereby improving the SR. Referring to Fig 2A, it is clear that, under the same blocklength, the performance of the SPC-NOMA VLC/RF scheme is better than that of the SPC-NOMA VLC scheme. This is due to the fact that, when an error occurs in decoding the VLC signal, the designed scheme can promptly use the RF network to provide the UE with auxiliary service. This mechanism improves the SR. Moreover, Fig 2B shows that the performance of the SPC-NOMA VLC scheme is better than that of the SPC-OMA VLC. This finding is due to the NOMA scheme that shares the entire blocklength with different power coefficients, while the OMA scheme should be divided into half of the blocklength, knowing that the NOMA scheme is more reliable.

In Fig 2A, “SPC-NOMA-VLC/RF” represents the resource optimization scheme based on SPC and NOMA technologies for VLC/RF networks, while “SPC-NOMA-VLC” denotes the resource optimization scheme based on the SPC and NOMA technologies for VLC network. In Fig 2B, “SPC-OMA-VLC” is the resource optimization scheme based on the SPC and OMA technologies for VLC network.

**Fig 3:**
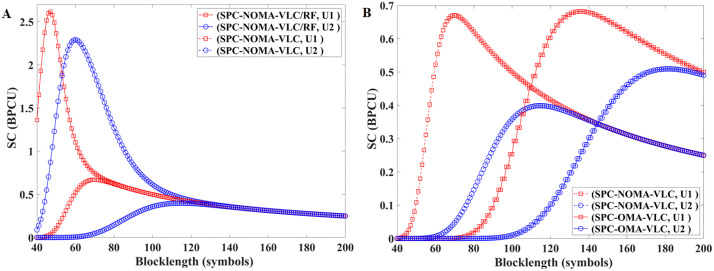
The impact of blocklength on the SC under different solutions. (A) Different scenarios. (B) Different technologies.

Fig 3 shows the impact of different schemes on UE service capabilities, where the parameter values are the same as those selected in Fig 2. Referring to Fig 3, when the blocklength *L* increases, the SC increases first and then decreases. This is because when the blocklength *L* gets longer, both the probability of successful communication and the SC will be bigger. However, when the blocklength *L* increases to a certain extent, the probability of successful communication hardly increases anymore, whereas the UE transmission rate starts to gradually decrease, and the SC begins to degrade after reaching the peak. As shown in Fig 3A, the SPC-NOMA VLC/RF scheme can achieve greater SC with a shorter blocklength compared to the scheme without RF assistance. Moreover, Fig 3B shows that the VLC scheme adopting NOMA technology can reach the SC peak more quickly with a shorter blocklength compared to the scheme using OMA technology. A short blocklength could better satisfy the communication equipment with low-latency requirements.

To sum up, when the blocklength is short, the VLC/RF scheme with NOMA technology is more suitable to ensure data transmission with high-reliability requirements compared with other schemes. For a clearer demonstration of the proposed scheme superiority in maximizing the SC, the optimal values extracted from Fig 3 are summarized in the Table 2. The largest values of the table are highlighted in bold. As shown in the Table 2, the SPC-NOMA-VLC/RF scheme outperforms both SPC-NOMA-VLC and SPC-OMA-VLC in supporting high capacity transmission under short blocklength conditions.

**Table 2:** Performance Comparison of Different Resource Optimization Schemes.

scheme value PI	U_1_		U_2_	
	*SC*_max_ (BPCU)	$L^{VLC*}$ (symbol)	*SC*_max_ (BPCU)	$L^{VLC*}$ (symbol)
SPC-NOMA-VLC/RF	**2.6160**	47	**2.2920**	60
SPC-NOMA-VLC	0.6707	70	0.3991	115
SPC-OMA-VLC	0.6825	135	0.5095	180

where PI is the abbreviation for “performance indicators”.

**Fig 4:**
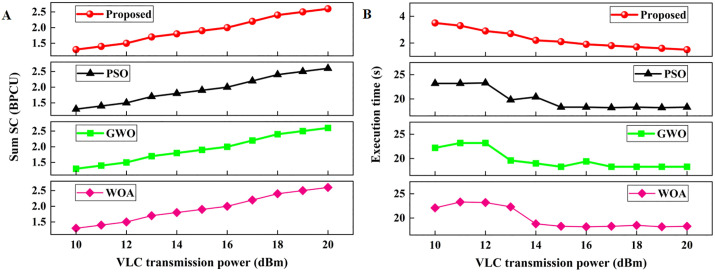
The impact of VLC transmission power on the sum SC and the execution time under different algorithms. (A) Sum SC (B) Execution time.

To validate the superiority of the proposed resource optimization algorithm compared with other methods, we solve the optimization problem utilizing typical heuristic-based approaches including Particle Swarm Optimization (PSO) [31], Grey Wolf Optimization (GWO) [32], and Whale Optimization Algorithm (WOA) [33]. The values of the population sizes and the maximum iteration number are set under the condition of ensuring the convergence of the three algorithms. The purpose is to avoid introducing unfair computational burdens due to excessively large parameter settings. The simulation is shown in Fig 4, and the results show that both the proposed algorithm and the comparison algorithms could obtain the same optimal solution. Subsequently, we analyze the operational efficiency of the algorithm. From Fig 4B, the proposed algorithm could settle the optimization problem requiring significantly less execution time, which proves the advantages of the proposed algorithm.

The data in Fig 4B are summarized in Table 3, which provides a more specific comparison of the efficiency between the proposed algorithm and heuristic-based algorithms. Table 3 presents the average execution time of each algorithm.

**Table 3:** Execution time of different algorithms.

Algorithm Value PAE	Average execution time (s)	Execution time ratio
Proposed	2.2936	—
WOA	19.9486	8.70
GWO	19.8185	8.64
PSO	19.9603	8.70

where PAE is the abbreviation for “Performance and efficiency”.

The proposed algorithm demonstrates an average execution time of approximately 2.3 s from Table 3, which is shorter than that of the other algorithms. Therefore, the proposed algorithm significantly enhances computational efficiency.

**Fig 5:**
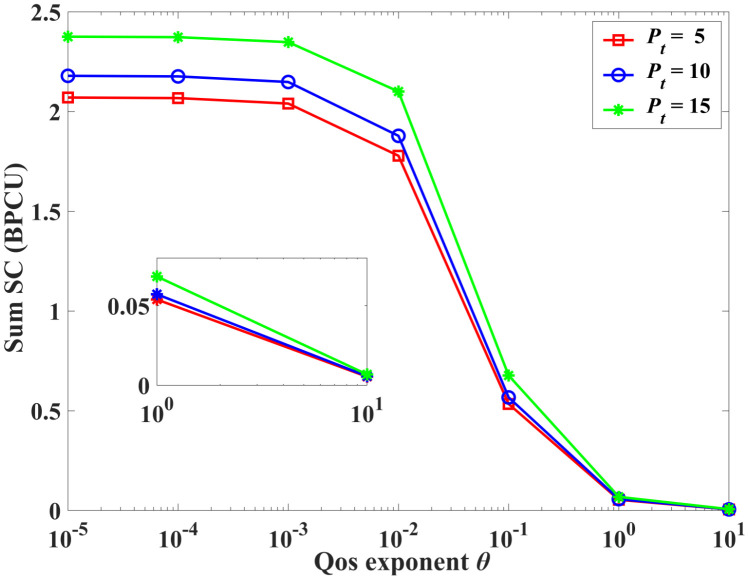
The impact of VLC transmission power on the sum SC under different QoS index.

Delay is a key metric in network performance evaluation and a fundamental QoS parameter, but it could merely reflect a certain aspect of service quality. From long-term stability perspective, the QoS index provides a more comprehensive reflection of delay performance. Therefore, we analyze the impact of the QoS index on the sum SC for the NOMA VLC/RF networks adopting SPC technology. As shown in Fig 5, when the QoS index is smaller (*θ* → 0), the SC is greater. This occurs because when the QoS index decreases, the QoS equipment requirements become stricter, the service required by the UE and network capacity increase. It can also be observed that, when the QoS index is unchanged, the greater the VLC transmission power, the bigger the network capacity. The reason is that when the VLC transmission power is set to a higher value, the UE will receive greater power, thereby increasing SC.

**Fig 6:**
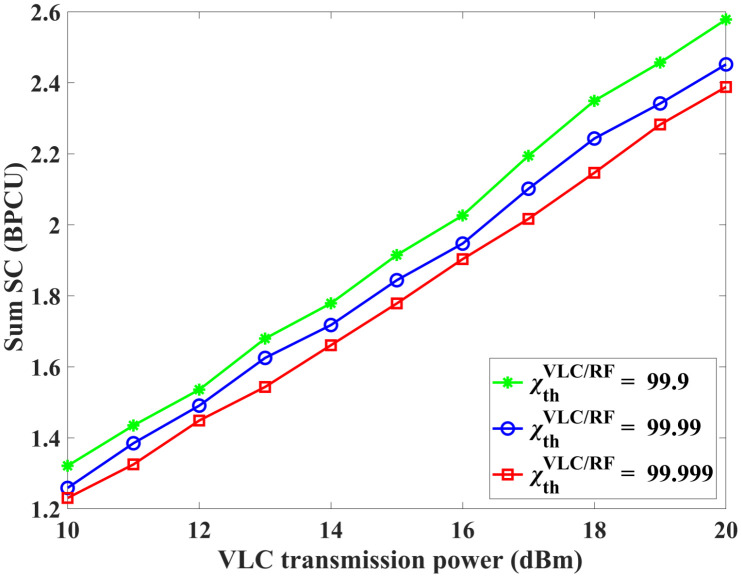
The impact of VLC transmission power on the sum SC under different SR.

Fig 6 presents the impact of target SR of VLC/RF networks, the RF transmission power, and the number of transmitted bits on the sum SC. The VLC transmission power is set to change from 10 dBm to 20 dBm, whereas $\chi_{th}^{VLC/RF}$ ranges are in the interval [99.9%, 99.99%, 99.999%]. As shown in Fig 6, a higher VLC transmission power results in a greater the sum SC. Simultaneously, under the same transmission power, a higher target SR leads to a lower sum SC. This is because when the target SR increases, the decoding error probability of UE_1_ has increased. The one way to enhance the SR is to increase the allocated power and blocklength. However, this reduces the service obtained by UE_2_, leading to an overall degradation in network performance.

**Fig 7:**
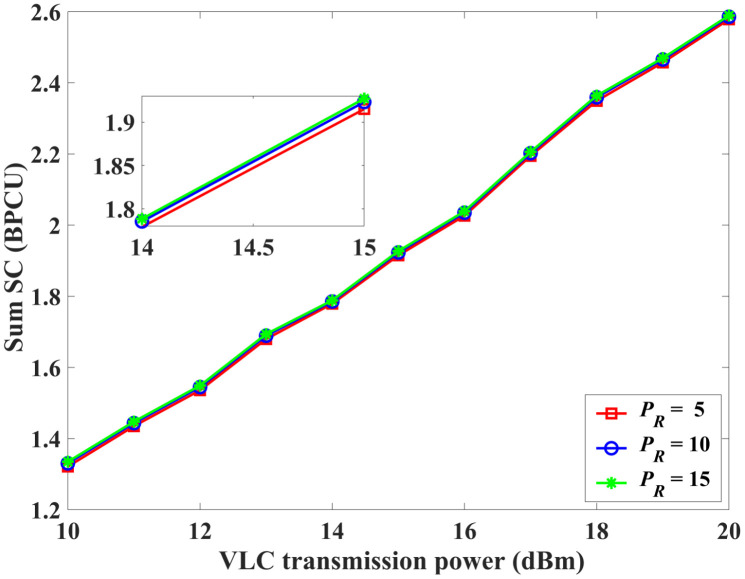
The impact of VLC transmission power on the sum SC under different RF transmission power.

Fig 7 investigates the impact of VLC transmission power on the sum SC under different RF transmission power. The RF transmission power *P*_*R*_ = [5 dBm, 10 dBm, 15 dBm] is set. It is clear that, when the VLC transmission power is fixed, the RF transmission power increases as well as the sum SC. This is because as the RF transmission power rises, the service provided by RF to the equipment becomes bigger. At the same time, it is clear that adjusting the RF transmission power affects slightly the sum SC. As the VLC transmission power increases, the network overall service capability varies substantially. The simulation results prove that the network performance mainly depends on the optimization of VLC parameters in the short packet transmission scenario.

**Fig 8:**
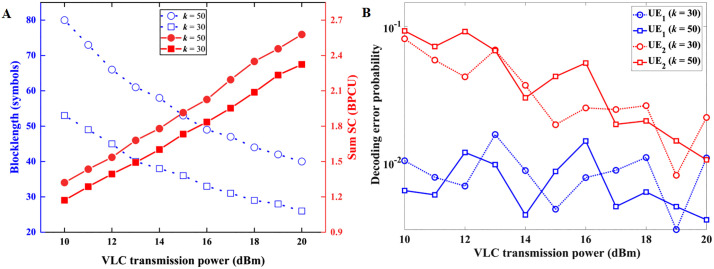
The impact of VLC transmission power and bits on system performance. (A) Blocklength and Sum SC. (B) Decoding error probability.

Fig 8 displays the impact of VLC transmission power on the blocklength, the sum SC, and decoding error probability. The number of transmission bits for the UE is set to *k* = 30 and *k* = 50, respectively. Referring to Fig 8A, it is clear that, under the same transmission bits, the transmission power and the blocklength present a negative correlation trend. As the VLC transmission power grows, the service rate rises, while the blocklength declines. Furthermore, a higher value of *k* results in a bigger blocklength and improves the sum SC. This is because when the number of transmission bits *k* increases, a larger blocklength is required to carry data packets, and more service should be supplied to process these data packets, thereby enhancing the network performance. Moreover, Fig 8B demonstrates that the decoding error probability is extremely low with the optimal solution, and the effectiveness of the scheme is validated.

**Fig 9:**
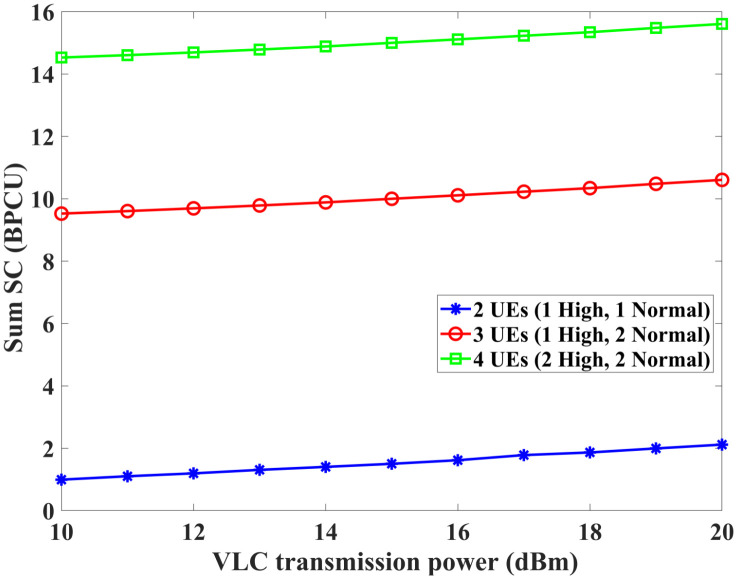
The impact of VLC transmission power on the sum SC under different user number.

Furthermore, to validate the extensibility of the algorithm in the multi-UE scenario, we evaluate the sum SC in three configuration scenarios: 2 UEs (1 high-reliability, 1 common), 3 UEs (1 high-reliability, 2 common), and 4 UEs (2 high-reliability, 2 common). Fig 9 illustrates the impact of the variation of VLC transmission power on the sum SC. As the VLC transmission power increases from 10 dBm to 20 dBm, the sum SC under all scenarios rises significantly. This improvement stems from the enhanced SINR at higher power levels, which boosts the service rate and reduces decoding error probability, thereby enhancing the sum SC. Moreover, it can be observed that for a given VLC transmission power, a larger number of UE leads to a higher the sum SC. Although incremental UE reduces the power allocated per UE, the system could serve more data packets simultaneously while still satisfying QoS requirements.

## 5. Model limitations and future extensions

We exhibit a resource optimization scheme joint blocklength control and power allocation, assuming the perfect SIC and CSI. Simulation results demonstrate that the proposed optimization scheme achieves greater SC with shorter blocklength while ensuring SR. It is worth noting that the proposed scheme holds applicability and could be further extended to analyze systems performance under the uncertain channel conditions. Specifically, the system model could be reconstructed by introducing residual interference factors and channel estimation errors for the scenarios of imperfect SIC and imperfect CSI. On this basis, the decoding error probability could be analyzed, and then the corresponding resource optimization problem also could be established. By applying the algorithm proposed, it is possible to obtain the optimal solution under the corresponding conditions. In the future work, we would further explore resource optimization problems under imperfect channel conditions to expand and deepen our research value in this field.

## 6. Conclusion

In this paper, we mainly introduce the resource optimization algorithm for MTC equipment with differentiated QoS requirements in NOMA VLC/RF networks. First, we construct an RF-aided NOMA VLC network model adopting the SPC technology. The successful transmission probability of the UE is derived and the SC of networks is evaluated under NOMA VLC/RF, NOMA VLC, and OMA VLC schemes. Then, under the constraints of UEs statistical delay QoS, SR, and transmission power, an optimization problem with SC maximization is built. We propose a resource optimization algorithm combining blocklength control and power allocation to settle this problem. Finally, the results show that, compared to the NOMA VLC and OMA VLC resource optimization schemes, the proposed NOMA VLC/RF resource optimization scheme could generate greater SC with a shorter blocklength and more suitable performance for high-reliability communication transmission. The presented scheme could provide a theoretical direction for resource optimization in real industrial environments, enabling the identification of optimization space under practical conditions and further guiding system design.

## Supporting information

S1 File Simulation Result Data. (XLSX)

## References

[1] Zirak M, Sedaghat Y, Yaghmaee Moghaddam MH. RPL-TSCH cross-layer design for improve quality of service in LLNs of IIoT. Ad Hoc Netw. 2025;174:103843. doi: 10.1016/J.ADHOC.2025.103843

[2] Pradhan A, Das S, Piran MJ. Blocklength optimization and power allocation for energy-efficient and secure URLLC in industrial IoT. IEEE Internet Things J. 2023;11(6):9420–31. doi: 10.1109/JIOT.2023.3324379

[3] Chang S, Huang N, Gong C, Li X-Y. Low-latency network slicing for VLC-based industrial internet of things: Superframe duration minimization and delay violation probability analysis. IEEE Internet Things J. 2023;10(18):16617–36. doi: 10.1109/jiot.2023.3268183

[4] Tong Y, Tang P, Zhang J, Liu S, Yin Y, Liu B, et al. Channel characteristics and link adaption for visible light communication in an industrial scenario. Sensors (Basel). 2023;23(7):3442. doi: 10.3390/s23073442 37050501 PMC10098709

[5] Zhou Y, Huang N, Xu Z. Outage and rate analysis for industrial internet of things in multitier visible light communication networks inside finite 3-D space. IEEE Internet Things J. 2024;11(11):20590–604. doi: 10.1109/jiot.2024.3371174

[6] Taneja A, Alqahtani A, Alqahtani N. Energy aware resource association in RIS assisted VLC-RF communication network. PLoS One. 2025;20(7):e0327467. doi: 10.1371/journal.pone.0327467 40694557 PMC12282862

[7] Yin L, Popoola WO, Wu X, Haas H. Performance evaluation of non-orthogonal multiple access in visible light communication. IEEE Trans Commun. 2016;64(12):5162–75. doi: 10.1109/tcomm.2016.2612195

[8] Chen C, Fu S, Jian X, Liu M, Deng X, Ding Z. NOMA for energy-efficient LiFi-enabled bidirectional IoT communication. IEEE Trans Commun. 2021;69(3):1693–706. doi: 10.1109/tcomm.2021.3051912

[9] Yapıcı Y, Güvenç I. NOMA for VLC downlink transmission with random receiver orientation. IEEE Transactions on Communications. 2019;67(8):5558–73. doi: 10.1109/TCOMM.2019.2914195

[10] Rallis KG, Papanikolaou VK, Diamantoulakis PD, Tegos SA, Dowhuszko AA, Khalighi M-A, et al. Energy efficient cooperative communications in aggregated VLC/RF networks with NOMA. IEEE Trans Commun. 2023;71(9):5408–19. doi: 10.1109/tcomm.2023.3292486

[11] Aghaei F, Eldeeb HB, Bariah L, Muhaidat S, Uysal M. Comparative characterization of indoor VLC and MMW communications via ray tracing simulations. IEEE Access. 2023;11:90345–57. doi: 10.1109/access.2023.3307186

[12] Aghaei F, Eldeeb HB, Uysal M. A comparative evaluation of propagation characteristics of vehicular VLC and MMW channels. IEEE Transactions on Vehicular Technology. 2023;73(1):4–13. doi: 10.1109/TVT.2023.3302991

[13] Zhao X, Sun JY. Secure reconfigurable intelligent surface aided heterogeneous VLC–RF cooperative NOMA networks. Opt Commun. 2022 May 15;511:127983. doi: 10.1016/j.optcom.2022.127983

[14] Han Y, Zhou X, Yang L, Li S. A Bipartite Matching Based User Pairing Scheme for Hybrid VLC-RF NOMA Systems. 2018 International Conference on Computing, Networking and Communications (ICNC), 2018. 10.1109/iccnc.2018.8390269

[15] Rallis KG, Papanikolaou VK, Diamantoulakis PD, Khalighi M-A, Karagiannidis GK. Energy Efficiency Maximization in Cooperative Hybrid VLC/RF Networks with NOMA. 2021 17th International Symposium on Wireless Communication Systems (ISWCS), 2021. 1–6. 10.1109/iswcs49558.2021.9562202

[16] Al Hammadi A, Sofotasios PC, Muhaidat S, Al-Qutayri M, Elgala H. Non-orthogonal multiple access for hybrid VLC-RF networks with imperfect channel state information. IEEE Trans Veh Technol. 2021;70(1):398–411. doi: 10.1109/tvt.2020.3044837

[17] Li Q, Shi L, Tang T, Xiong Z. User cooperation and link selection in Hybrid VLC/RF systems with NOMA. IEEE Wireless Commun Lett. 2024;13(4):979–83. doi: 10.1109/lwc.2024.3355097

[18] Eren T. Non-coherent short-packet communications: Novel z-domain user multiplexing. Digital Signal Processing. 2025;156:104777. doi: 10.1016/j.dsp.2024.104777

[19] Bao BQ, Thuc KX, Hiep PT. Short-packet communication for MIMO NOMA relay networks: BLER and AoI analysis. Int J Electron Commun. 2024;174:155035. doi: 10.1016/J.AEUE.2023.155035

[20] Yuan L, Du Q, Fang F. Performance analysis of full-duplex cooperative NOMA short-packet communications. IEEE Trans Veh Technol. 2022;71(12):13409–14. doi: 10.1109/tvt.2022.3199541

[21] Xia CL, Xiang ZW, Su BB, Liu HB, Meng J, Pan GF. RIS-NOMA-assisted short-packet communication with hardware impairments. IEEE Internet of Things Journal. 2023;11(2):2990–3002. doi: 10.1109/JIOT.2023.3294257

[22] Hammouda M, Peissig J. VLC Systems with Fixed-Rate Transmissions under Statistical Queueing Constraints. 2018 11th International Symposium on Communication Systems, Networks & Digital Signal Processing (CSNDSP), 2018. 1–6. 10.1109/csndsp.2018.8471833

[23] Dapeng Wu, Negi R. Effective capacity: A wireless link model for support of quality of service. IEEE Trans Wireless Commun. 2003;24(5):630–43. doi: 10.1109/twc.2003.814353

[24] Grubor J, Randel S, Langer K-D, Walewski JW. Broadband information broadcasting Using LED-based interior lighting. J Lightwave Technol. 2008;26(24):3883–92. doi: 10.1109/jlt.2008.928525

[25] Tran DD, Sharma SK, Chatzinotas S, Woungang I, Ottersten B. Short-packet communications for MIMO NOMA systems over Nakagami-m fading: BLER and minimum blocklength analysis. IEEE Transactions on Vehicular Technology. 2021;70(4):3583–98. doi: 10.1109/TVT.2021.3066367

[26] Le NP, Le KN. Uplink NOMA short-packet communications with residual hardware impairments and channel estimation errors. IEEE Transactions on Vehicular Technology. 2022;71(4):4057–72. doi: 10.1109/TVT.2022.3148124

[27] Qian L, Chi X, Zhao L, Obeed M, Chaaban A. User-centric secure cell formation for visible light networks with statistical delay guarantees. IEEE Trans Wireless Commun. 2021;20(3):1831–46. doi: 10.1109/twc.2020.3036907

[28] Xiao Y, Diamantoulakis PD, Fang Z, Ma Z, Hao L, Karagiannidis GK. Hybrid Lightwave/RF Cooperative NOMA Networks. IEEE Trans Wireless Commun. 2020;19(2):1154–66. doi: 10.1109/twc.2019.2951401

[29] Kemeng Yang, Gondal I, Bin Qiu. Multi-dimensional adaptive sinr based vertical handoff for heterogeneous wireless networks. IEEE Commun Lett. 2008;12(6):438–40. doi: 10.1109/lcomm.2008.080216

[30] Ha-Xuan S, Quynh TPT, Le-Tran M, Anh VT. Improving the Reliability of Short Packet Communications in VLC Systems. 2023 International Conference on Advanced Technologies for Communications (ATC), 2023. 468–73. 10.1109/atc58710.2023.10318860

[31] Kennedy J, Eberhart R. Particle swarm optimization. Proceedings of ICNN’95 - International Conference on Neural Networks, Perth, WA, Australia, 1995. 10.1109/ICNN.1995.488968

[32] Mirjalili S, Mirjalili SM, Lewis A. Grey wolf optimizer. Adv Eng Softw. 2014;69:46–61. 10.1016/j.advengsoft.2013.12.007

[33] Mirjalili S, Lewis A. The whale optimization algorithm. Adv Eng Softw. 2016;95:51–67. doi: 10.1016/j.advengsoft.2016.01.008

